# Cellular Responses at the Zirconia Dental Implant Interface: A Comprehensive Review

**DOI:** 10.3390/jfb17080372

**Published:** 2026-08-01

**Authors:** Marija S. Milic, Jelena Simonovic, Vladimir S. Todorovic

**Affiliations:** 1 General and Oral Physiology Department, School of Dental Medicine, University of Belgrade, 11000 Belgrade, Serbia; 2 General and Oral Histology and Embryology Department, School of Dental Medicine, University of Belgrade, 11000 Belgrade, Serbia; jelena.simonovic@stomf.bg.ac.rs; 3 School of Dental Medicine, University of Belgrade, 11000 Belgrade, Serbia; vladimir.todorovic@stomf.bg.ac.rs

**Keywords:** zirconia, dental implants, implant surface, cell cultures, osteoblasts, fibroblasts, macrophages, epithelial cells, mesenchymal stem cells

## Abstract

Titanium remains the gold standard in dental implantology; however, its clinical drawbacks, such as hypersensitivity reactions, metallic particle release, and aesthetically compromising discoloration, have driven interest in metal-free alternatives. Yttria-stabilized tetragonal zirconia polycrystal (Y-TZP) has emerged as a promising bioceramic candidate, offering favorable aesthetics, mechanical strength, and reduced bacterial plaque affinity. Its pristine surface is nonetheless bioinert, prompting extensive research into surface modification strategies, including sandblasting, acid-etching, femtosecond laser texturing, and bioactive coatings, to enhance osteoconductivity. This comprehensive narrative review synthesizes in vitro evidence on the behavior of key host cell populations—macrophages, mesenchymal stem cells, osteoblasts, fibroblasts, and epithelial cells in response to Y-TZP surface and its modifications, relevant to osseointegration and soft-tissue sealing. A literature search was conducted across PubMed, Scopus, Web of Science, and the Cochrane Library, supplemented by Google Scholar, concluding in March 2026. By integrating findings across multiple cell lineages, this review aims to clarify how engineered zirconia topographies modulate cell-specific pathways, thereby informing the development of next-generation implants optimized for biological integration.

## 1. Introduction

Titanium (Ti) has traditionally held the position of the gold standard in dental implantology, with high long-term survival rates [1]. However, Ti presents certain clinical limitations, including potential hypersensitivity reactions, the accumulation of metallic particles in surrounding tissues, and greyish discoloration that severely compromises aesthetics, particularly in patients with thin peri-implant mucosa [2,3].

The biological consequences of titanium particle release have been increasingly documented in recent years. A tribocorrosive interplay between biofilm adhesion, chemical contact, and mechanical wear has been proposed to drive the degradation of titanium implant surfaces, resulting in the release of metal ions and nanoparticles into peri-implant tissues. An umbrella review by Di Spirito et al. estimated an overall prevalence of 16.9% of cases presenting with lesions potentially attributable to titanium-derived nanoparticles, encompassing lesions resembling peri-implant mucositis and peri-implantitis (55.17%), reactive exophytic lesions of the peri-implant mucosa (17.22%), and orofacial hypersensitivity reactions (24.12%) [4]. These findings underscore the growing clinical awareness of adverse biological reactions potentially linked to titanium-derived particles, supporting a rationale for the development of metal-free ceramic alternatives.

These drawbacks have catalyzed the demand for metal-free alternatives, positioning zirconia (ZrO_2_), specifically yttria-stabilized tetragonal zirconia polycrystals (Y-TZP) as a highly promising bioceramic [5]. Y-TZP has established itself as a highly competitive ceramic biomaterial in contemporary dental implantology owing to its tooth-like aesthetics, exceptional biocompatibility, mechanical properties, and a significantly reduced affinity for bacterial plaque, which collectively mitigate the risk of peri-implant inflammatory diseases [6].

At the materials science level, pure ZrO_2_ exhibits a polymorphic structure existing in monoclinic, tetragonal, and cubic phases depending on the temperature [7]. By doping pure zirconia with stabilizing oxides, such as 3 mol% yttria, the metastable tetragonal phase is successfully retained at room temperature, yielding Y-TZP [8]. This structural configuration leverages a unique martensitic phase transformation toughening mechanism: upon encountering localized stress, the tetragonal phase transforms into the monoclinic phase, triggering a volume expansion that actively compresses and arrests propagating micro-cracks [9]. Consequently, Y-TZP is characterized by low porosity, high density, high flexural strength, and exceptional fracture toughness [10]. Despite these remarkable mechanical properties, pristine Y-TZP is susceptible to low-temperature degradation (LTD) or aging, a moisture-induced phase transformation that can compromise its long-term mechanical stability [11,12].

While the mechanical profile of Y-TZP is outstanding, its pristine surface is inherently bioinert. Untreated zirconia exhibits limited protein adsorption and cellular attachment in laboratory settings, which can lead to suboptimal osseointegration [13,14]. To overcome this fundamental limitation, a wide range of surface modification strategies has been extensively documented in cell culture studies to enhance bioactivity. Physical and subtractive treatments, such as sandblasting, acid-etching, and advanced femtosecond laser texturing, are widely applied to generate hierarchical micro- and nano-topographies that elevate surface energy and wettability [15,16]. Furthermore, chemical functionalizations and bioactive coatings, including calcium phosphate (CaP), hydroxyapatite (HA), biomimetic polydopamine (PDA), and cell-adhesion peptides (e.g., RGD motifs), are engineered to mimic the native extracellular matrix (ECM) and confer enhanced osteoconductive properties in vitro [14,17].

**Figure 1:**
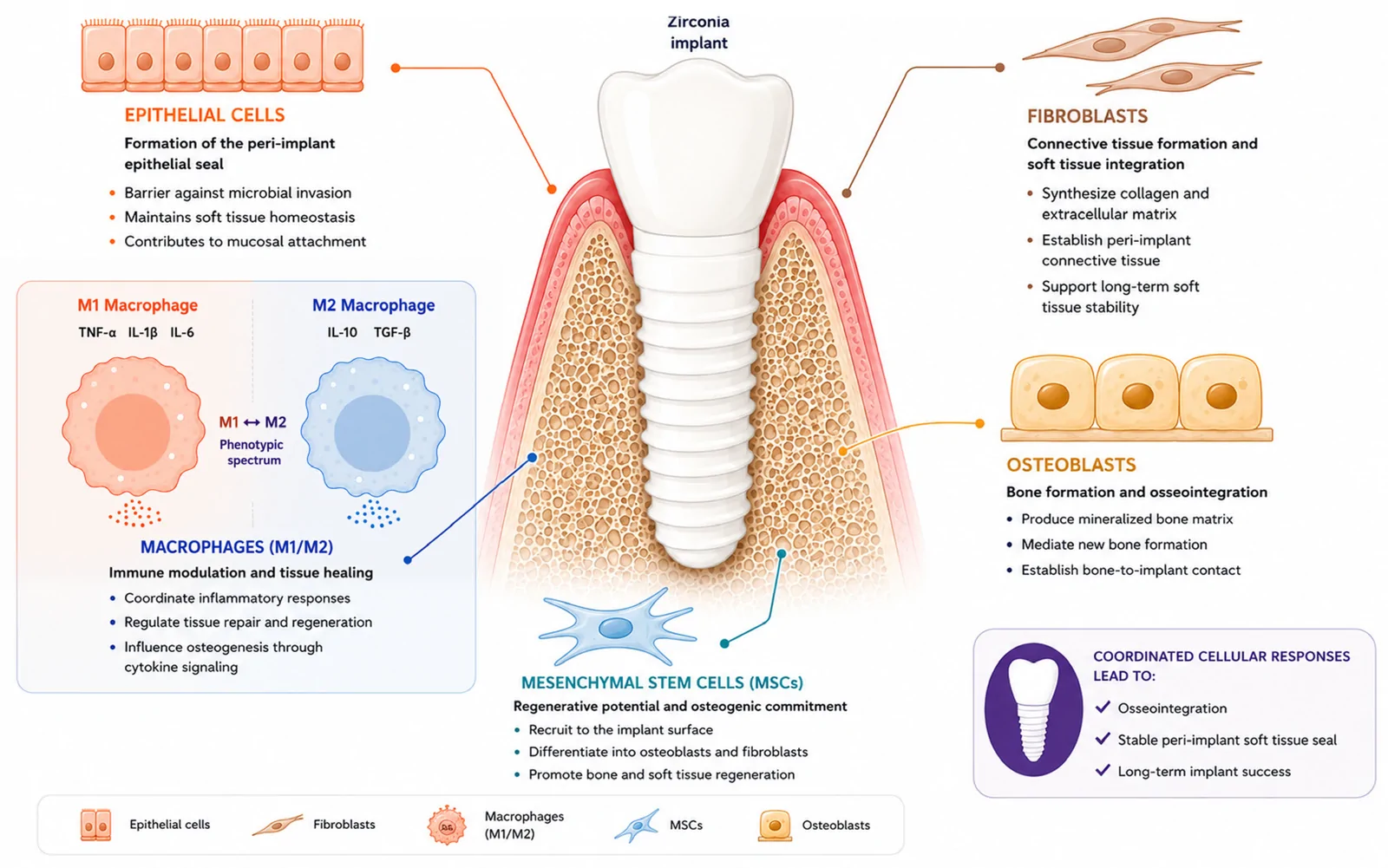
Schematic overview of key host cell responses at the zirconia implant–tissue interface, encompassing macrophages, mesenchymal stem cells, osteoblasts, fibroblasts, and epithelial cells relevant to osseointegration and soft-tissue sealing.

The ultimate success of a dental implant relies on the complex host–material interactions occurring at the biological interface (Figure 1), which are simulated using isolated cell lines.

The early modulation of immune cells, particularly the polarization of macrophages from a pro-inflammatory (M1) to a pro-regenerative, wound-healing (M2) phenotype, is essential for promoting a microenvironment favorable for implant integration [18]. Furthermore, the recruitment and differentiation of mesenchymal stem cells (MSCs) are critical for establishing the regenerative pathways. In strictly controlled in vitro environments, MSCs exhibit excellent adhesion and early osteogenic differentiation when interacting with biofunctionalized zirconia, particularly surfaces coated with calcium phosphate or specialized nanoparticles [19,20]. Following early immune modulation by macrophages and the recruitment of MSCs, osteoblasts must successfully attach, mature and proliferate on micro-rough topographies to drive the final stages of osseointegration [21].

In the transmucosal region, the establishment of a resilient biological seal relies heavily on the response of epithelial cells and gingival fibroblasts [22,23]. Epithelial cells must rapidly migrate, adhere via hemidesmosomes, and proliferate to form a functional physical barrier [24]. Unlike osteoblasts that thrive on micro-rough topographies, isolated epithelial cells in culture often demonstrate superior attachment and spreading on smoother or specifically nano-engineered zirconia surfaces [21,25].

A thorough understanding of these cell-specific responses to modified topographies remains the critical prerequisite for the successful biological integration and long-term clinical outcome of zirconia-based dental implants. Existing reviews predominantly approach zirconia from a materials science or surface engineering perspective; however, a comprehensive synthesis centered on cell-specific biological responses across the entire peri-implant healing cascade remains lacking. This review addresses that gap by evaluating the behavior of key mammalian host cell populations (macrophages, mesenchymal stem cells, osteoblasts, fibroblasts, and epithelial cells) in response to distinct surface modifications applied to Y-TZP implants. By synthesizing current in vitro evidence, this review aims to elucidate how engineered zirconia surfaces modulate critical cellular behavior at the micro- and nanoscale, offering a translational perspective for the development of next-generation biomaterials optimized for osseointegration and soft tissue sealing.

## 2. Materials and Methods

### 2.1. Study Design

This paper was designed as a comprehensive narrative review of in vitro studies evaluating the cellular responses to Y-TZP dental implant material. Given its narrative design, systematic-review procedures based on the PRISMA framework were not considered applicable. Relevant studies were selected on the basis of their scientific relevance to the review objectives and expert judgment, without formal screening counts or risk-of-bias assessment. By integrating findings from cell culture investigations across multiple cell lineages, this work aims to provide an updated and synthesized perspective on the biological interactions between Y-TZP surfaces and host cells relevant to dental implant osseointegration and soft-tissue integration.

### 2.2. Literature Search

To identify the relevant literature, a targeted literature search was conducted to identify representative in vitro cell culture studies published in English in peer-reviewed journals. The primary databases searched included PubMed, Scopus, Web of Science, and the Cochrane Library, with Google Scholar used as a supplementary source. The search strategy combined controlled vocabulary (e.g., MeSH terms) and free-text keywords targeting the implant material, clinical application, and cell type, tailored to the specific syntactical requirements of each database. The search terms included: “zirconium oxide”, “zirconia”, “yttria stabilized zirconia”, “Y-TZP”, “dental implants”, “dental implant*”, “implant surface*”,”implant material*”, “osteoblasts”, “osteoblast*”, “osteocyte*”, “fibroblasts”, “fibroblast”, “gingival fibroblast*”, “mesenchymal stem cells”, “mesenchymal stem cell*”, “MSC”, “MSCs”, “macrophages”, “macrophage*”, “monocyte*”, “polarization”, “epithelial cells”, “epithelial cell*”, “keratinocyte*”, “epithelium”. Studies were included if they were in vitro cell culture investigations evaluating Y-TZP as a dental implant material and reported quantitative or qualitative outcomes related to cellular responses. Studies were generally excluded if they were non-peer-reviewed, focused exclusively on mechanical or physicochemical properties without cellular outcomes, or investigated only in vivo or ex vivo models, consistent with the review’s emphasis on in vitro cellular biology. Given the narrative nature of this review, no formal risk-of-bias or quality-scoring instrument was applied. However, the included studies varied considerably in experimental design, spanning immortalized cell lines, primary human cells, and murine-derived models, with differing numbers of biological/technical replicates and statistical approaches. The initial literature search was completed in March 2026 and subsequently updated on 15 June 2026, prior to submission to capture any newly published literature.

## 3. Results

### 3.1. Macrophages

The initial events following implantation are governed by blood–surface biological interactions, where the “Vroman effect” dictates the sequential adsorption of blood proteins, directly influencing subsequent cellular behaviors [21]. The concept of the “race for the surface” highlights the critical competition between tissue cell integration and bacterial colonization at this interface [13,26]. The success of soft and hard tissue integration is determined by the early immune-mediated foreign body reaction triggered upon implantation [27]. Macrophages, derived from the monocyte lineage, are among the very first cells to infiltrate the peri-implant microenvironment and orchestrate the inflammatory and tissue-repair cascades [28,29]. Displaying high phenotypic plasticity, macrophages polarize into “classically activated” M1 macrophages (secreting pro-inflammatory cytokines such as TNF-α, IL-6, and IL-1β) or “alternatively activated” M2 macrophages (releasing anti-inflammatory factors such as IL-10 and TGF-β) [30,31,32]. A timely transition from the M1 to the M2 phase, a process termed osteoimmunomodulation, is critical to prevent prolonged inflammation and facilitate stable integration [33].

#### 3.1.1. Cell Lines Utilized in Current Evidence

To accurately evaluate the osteoimmunomodulatory potential of modified zirconia surfaces in vitro, researchers have utilized specific immune cell models. Current evidence relies heavily on the murine-derived macrophage cell line RAW 264.7 [34,35,36,37]. Human models are represented through THP-1 monocytes differentiated into macrophages [38], and primary human macrophages [39].

#### 3.1.2. Initial Adhesion and Morphological Dynamics

Physical modifications (such as machining, polishing, sandblasting, and acid-etching) significantly alter cellular behavior by creating specific macro-, micro-, and nano-topographies [40]. Early macrophage adhesion and spreading are heavily dictated by these topographical changes. Siddiqui et al. (2019) [39] demonstrated that human macrophages undergo distinct morphological adaptations based on topography: on smooth, polished Y-TZP surfaces (Sa ~ 0.08–0.22 µm), macrophages appeared highly spread, whereas on rougher acid-etched and sandblasted surfaces (Sa ~ 0.53–1.20 µm), they adopted a spindle-like configuration with a greater distribution of actin filaments attached to the surface at different angles [39]. Conversely, Wang et al. (2019) [37] noted that standard zirconia (ZLA) surfaces can actually decrease macrophage cell attachment when compared to titanium [37].

At the nanoscale, smooth topographies similarly enhance early attachment. Tian et al. (2024) [35] demonstrated that traditional 3 mol% Y-TZP inherently provides favorable nanoscale smoothness compared to titanium (Ra: ~59 nm vs. ~198 nm), which significantly enhanced early macrophage spreading [35]. Similarly, Wu et al. (2021) [36] highlighted that RAW 264.7 macrophages favor early attachment and higher proliferation on smooth, blank-machined nanostructured zirconia (BMZ; Ra = 7.6 ± 0.3 nm, water contact angle = 85.55°) compared to rougher self-glazed zirconia (SGZ; Ra = 49.3 ± 3.5 nm, water contact angle = 61.19°) [36].

#### 3.1.3. Impact of Specific Surface Modifications on Macrophage Behavior

**Topographical and Nanostructural Modifications:** While topographical micro-roughness is often used to enhance osteoblast mechanical interlocking, it tends to provoke a pro-inflammatory M1 response across different materials, including pure titanium, titanium-zirconium, and zirconia (ZLA) [37]. Modifying topography at the nanoscale, however, can effectively guide this behavior. Wu demonstrated that rough SGZ surfaces significantly upregulated pro-inflammatory M1 markers (IL-1β, iNOS, TNF-α), while smoother BMZ surfaces directed polarization toward the anti-inflammatory M2 phenotype, marked by higher IL-10 expression [36]. Furthermore, it was demonstrated that the inherent nanoscale smoothness of traditional 3Y-TZP successfully shifts polarization toward the M2 phenotype, downregulating pro-inflammatory genes (IL-6 and TNF-α) and upregulating pro-repair genes (IL-10 and TGF-β) when compared to rougher titanium surfaces [35].

**Surface Hydrophilicity and Wettability:** High surface free energy and wettability are considered essential for resolving inflammation [23]. To combat the pro-inflammatory tendencies of micro-roughness, authors have identified surface hydrophilicity as a prominent mechanism to ‘rescue’ cellular behavior. Modifying roughened pure titanium and titanium-zirconium (Ti-Zr) alloy surfaces to become highly hydrophilic successfully rescues the topography-induced pro-inflammatory response, heavily suppressing TNF-α and elevating IL-10 production [37]. However, on specific nanostructured zirconia surfaces, physical topography may exert a stronger influence on cell behavior than wettability. In Wu’s experimental model, the roughest zirconia surface (SGZ) exhibited the most favorable hydrophilicity, yet it still induced the highest pro-inflammatory M1 response, whereas the smoothest nanostructured surface (BMZ) directed M2 polarization despite being the most hydrophobic surface tested [36].

**Biofunctional Coatings:** Advanced organic and inorganic coatings are increasingly applied to zirconia to enhance biocompatibility and actively suppress implant-induced immune inflammation [17]. Cheng demonstrated that fullerene (C60) films on zirconia drastically alter the cytokine profile of THP-1 macrophages. Following a lipopolysaccharide (LPS) challenge, macrophages on C60-functionalized zirconia secreted significantly lower levels of key pro-inflammatory cytokines, specifically IL-1β and TNF-α, yielding a pronounced anti-inflammatory effect [38]. Regarding IL-6, C60 surface functionalization on zirconia showed no overall benefit or detriment for IL-6 protein production, although a downregulation in its gene expression was observed [38]. This immunomodulatory success was highly material-specific; applying the exact same C60 coating to titanium alloy (Ti-6Al-4V) successfully lowered TNF-α and exhibited the greatest drop in IL-6 concentration, but simultaneously triggered a significant increase in IL-1β secretion over time [38]. Finally, subjecting these C60 coatings to mechanical friction (tribological loading) diminished their anti-inflammatory capabilities by triggering higher IL-1β secretion, suggesting C60 functionalization is most promising when applied specifically to the smooth transmucosal abutment rather than bone-anchored threads [38].

**Time-Controlled Bilayer “Smart” Coatings:** To overcome these limitations and address both early acute inflammation and long-term bone integration, researchers are developing “smart” multi-stage coatings. Song and coworkers [34] engineered a time-controlled bilayer “sandwich” structure on zirconia by combining a femtosecond laser-etched micro/nano-topography with cerium dioxide nanoparticles (CeO_2_ NPs) modified with polydopamine (PDA) [34]. This design features an outer layer for the rapid release of CeO_2_ to attenuate early acute inflammation, while the inner layer continuously releases lower doses of CeO_2_ over several weeks to synergize with the laser-etched topography and promote long-term osseointegration [34]. When challenged with LPS, this composite coating directly inhibited the TLR4/MyD88/NF-κB signaling pathway, shifting macrophages away from the pro-inflammatory M1 phenotype and strongly inducing the pro-repair M2 phenotype [34]. Macrophages cultured on this coating demonstrated excellent reactive oxygen species (ROS) scavenging capacity and secreted significantly lower levels of pro-inflammatory cytokines (IL-1β and IL-6) alongside markedly elevated levels of the anti-inflammatory cytokine IL-10 [34].

#### 3.1.4. Key Remarks

The early immune response to zirconia implants determines the trajectory of peri-implant healing, necessitating surface modifications that actively drive macrophage polarization from the M1 to the M2 phenotype. While conventional micro-roughness successfully promotes the mechanical interlocking required for osteoblast anchorage, it paradoxically triggers an acute M1 inflammatory cascade. To resolve this inherent topographical conflict, engineering strategies should decouple macroscopic retention from microscopic inflammation.

Evidence suggests this could be achievable through precise nanostructural refinement, where the inherent nanoscale smoothness of zirconia can override even hydrophobic tendencies to dictate M2 polarization, or through advanced “smart” functionalization. By deploying tailored biochemical interventions, such as fullerene films or time-controlled cerium dioxide bilayer coatings, it is possible to actively silence local inflammatory signaling pathways (such as TLR4/NF-κB) independent of the underlying physical roughness. Ultimately, the successful osteoimmunomodulation of zirconia requires a multifaceted approach where bulk topography establishes physical anchorage, while advanced chemical and nanostructural modifications actively pacify the local immune microenvironment to prevent chronic fibrous encapsulation and ensure long-term osseointegration.

From a critical standpoint, the available evidence on macrophage responses to zirconia remains limited in both quantity and methodological consistency. The majority of studies rely on murine RAW 264.7 cells, which differ substantially from primary human macrophages in their polarization kinetics and cytokine profiles, limiting direct clinical extrapolation. Experimental conditions, such as LPS concentrations used to simulate inflammation, culture duration, and surface characterization methods vary considerably across studies, precluding reliable quantitative comparisons. Although nanostructured and functionalized zirconia surfaces show promising osteoimmunomodulatory potential, to our knowledge, no clinical study has directly demonstrated that surface-induced M2 polarization accelerates or enhances the osseointegration of zirconia dental implants in a clinical setting. The evidence base, though mechanistically informative, must therefore be interpreted with caution until validated by in vivo models and prospective clinical data.

### 3.2. Mesenchymal Stem Cells

Mesenchymal stem cells are multipotent stem cells that are considered among the most important cell populations for successful osseointegration and osteogenic differentiation. They are normally present in tissues of mesodermal origin, such as bone. Since MSCs possess potential for osteogenic differentiation, their early response is regarded as a key determinant of the long-term success of dental implants [21].

#### 3.2.1. Cell Lines Utilized in Current Evidence

Biological behavior of zirconia-based materials, specifically Y-TZP, was primarily investigated on human bone marrow-derived mesenchymal stem cells (hMSCs) either primary isolated [41] or commercially available cell lines (Lonza, ATCC and Zen-Bio) [42,43,44], and human adipose-derived mesenchymal stem cells (hADMSCs) [45], while in one manuscript the type of hMSCs used in experiment was not specified [46].

#### 3.2.2. Adhesion, Spreading, and Morphological Dynamics

The initial adhesion and spreading of cells on zirconia surfaces are mostly influenced by surface topography. While polished zirconia often results in rounded cell morphologies with a poorly developed cytoskeleton, increasing surface roughness improves cell area and spreading by providing more contact surface for anchorage [44,46,47]. Specifically, focal adhesions (FAs), the protein complexes that link the cytoskeleton to the surface layer, are modulated by laser-induced nanotopography, which enhances integrin clustering and the formation of nascent focal complexes associated with strong cell-surface interactions [44,47,48]. Through the phenomenon of contact guidance, microgrooved patterns result in a shape change, causing cells to elongate and align parallel to the grooves [43,44]. Research using roughness gradients has further established that complexity of cell shape is linearly correlated with nanoroughness parameters, whereas overall cell area is more sensitive to microroughness [41].

#### 3.2.3. Proliferation vs. Osteogenic Differentiation

Surface modifications are used to stimulate both the cell proliferation and differentiation. Surface topographies with hierarchical organization, such as those that are results of sandblasting followed by acid etching (SLA-like surfaces), have been shown to significantly enhance the proliferation of hMSCs, alkaline phosphatase (ALP) activity, and the expression of osteogenic marker genes, including RUNX family transcription factor 2 (*RUNX2*), and Sp7 transcription factor (*SP7*) which encodes the osterix transcription factor [42]. Laser-textured surfaces, particularly those with a 10 μm periodicity, can upregulate the expression of bone-related genes. However, the most significant effects on differentiation of hMSCs are observed when topography modification is combined with biochemical functionalization; for instance, the addition of BMP-2-derived DWIVA peptides can result in a 2.5-fold upregulation of ALP activity and higher level of mineral matrix deposition independent of the topography [43].

#### 3.2.4. Impact of Specific Surface Modifications on Mesenchymal Stem Cells Behavior

**Topographical and Structural Modifications:** Procedures such as sandblasting and grinding are standard for reaching an optimal microroughness range (Sa ~ 1.0–2.0 μm) for bone response [48]. However, while these treatments enhance osseointegration, aggressive mechanical protocols can introduce surface imperfections that jeopardize the material’s hydrothermal and mechanical stability [48]. Hirano et al. (2015) [42] compared the osteogenic effect of sandblasted and acid-etched surfaces to a control group with a mirror-polished surface, and their results showed significantly higher ALP activity and cell proliferation in the acid-etched group, demonstrating that surface microroughness is a potent mechanical stimulus for the osteogenic commitment of hMSCs [42].

**Laser Ablation and Micro-Patterns:** Femtosecond (ultrashort pulsed) lasers appear to be superior to nanosecond lasers as they remove material by means of cold ablation, thus minimizing thermal damage, microcracks, and phase alterations [43,44,46]. Linear patterns are shown to favor unidirectional migration, doubling or tripling migration length parallel to the grooves, while grid patterns facilitate migration in multiple directions and enhanced cell spreading [43,44].

**Macroscopic Porosity and Additive Manufacturing:** Scaffolds featuring grooves and holes in the rod section of Y-TZP support the growth of hADMSCs within the material’s porosity, demonstrating biocompatibility and negligible cytotoxicity [45].

**Surface Hydrophilicity and Wettability:** While 3Y-TZP is inherently hydrophilic, laser-induced surface patterns often exhibit more hydrophobic behavior (Cassie-Baxter state). However, by tuning the depth and width of grooves, wettability can be adjusted either to promote cell adhesion or to reduce bacterial attachment via superhydrophobicity [44,48].

**Biofunctional Coatings:** Dual-peptidic platforms containing RGD (adhesive) and DWIVA (osteogenic) motifs, anchored by L-DOPA residues, maximize biological activity. This biochemical approach allows for the specific recruitment of integrins and BMP receptors, promoting osteogenic differentiation even on polished surfaces [43].

**The Inflammatory Microenvironment and Pathogenic Challenge:** The clinical success of zirconia depends on the “race for the surface” between host cells and bacteria [44,46]. Low-periodicity patterns (~3 μm) are particularly effective since they disrupt the aggregation of *Staphylococcus aureus* and mechanically trap *Pseudomonas aeruginosa*, thereby significantly reducing colonization. Co-culture experiments confirm that these patterned surfaces protect hMSCs from bacterial-induced necrosis, preserving high cell viability (up to 99%) in infected environments [44].

#### 3.2.5. Key Remarks

The collective evidence demonstrates that cell behavior on zirconia is governed by a multiscale structural hierarchy. Zirconia surfaces with appropriate topographical complexity appear to support better early cellular affinity and differentiation than titanium or smooth ceramic variants, as the rougher texture increases the available area for protein adsorption and focal adhesion formation. Microtopography acts as a primary spatial controller for macroscopic orientation and migration through physical confinement. On the other hand, nanotopography acts as a local cue regulating focal adhesion assembly and mechanotransduction. The integration of USP laser patterning with post-laser annealing represents the promising approach for generating cell-inductive and antibacterial surfaces without sacrificing the mechanical integrity or aesthetic quality of the zirconia material. Future strategies should combine these precise topographical cues with multifunctional biochemical platforms to optimize the biological response at different implant levels, including bone and soft tissue.

From the critical point of view, the MSCs literature on zirconia is characterized by considerable heterogeneity in cell sources, ranging from bone marrow-derived to adipose-derived MSCs, and from primary isolates to commercial lines. Each of these types exhibit distinct differentiation potentials and surface sensitivity profiles that complicate cross-study comparisons. The most consistently supported finding is that hierarchical micro/nano-topographies generated by femtosecond laser patterning may offer an advantage over conventional sandblasted or acid-etched surfaces in terms of directional migration and early osteogenic commitment. However, the majority of studies evaluate single surface variables in isolation, and few employ clinically relevant loading conditions or co-culture systems that would better approximate the in vivo peri-implant environment. Although statistically meaningful, the clinical significance of observed differences in ALP activity or *RUNX2* expression has not yet been validated against implant survival or bone-to-implant contact data in human trials.

### 3.3. Osteoblasts

Following the initial immune-mediated inflammatory phase, successful osseointegration depends on the subsequent recruitment, adhesion, and maturation of bone-forming cells. Osteoblasts must proliferate across the peri-implant microenvironment and undergo a timely osteogenic differentiation to secrete the mineralized extracellular matrix (hydroxyapatite) that firmly anchors the implant into the alveolar bone [49,50]. To overcome the inherent biological inertness of conventional zirconia and enhance its osteoconductive capabilities, various surface engineering protocols have been developed [15].

#### 3.3.1. Cell Lines Utilized in Current Evidence

To elucidate the mechanisms of osseointegration, various established in vitro models have been employed to evaluate the osteoblastic response to ZrO_2_ surfaces. The most widely utilized cell lines include murine pre-osteoblast MC3T3-E1 cells [38,39,51,52,53,54,55,56,57,58,59,60,61,62,63], human fetal osteoblasts such as hFOB 1.19 [64,65,66,67], human primary osteoblasts [68,69], as well as osteoblasts derived from redundant alveolar bone [70]. Osteosarcoma-derived cell lines, including MG-63, SAOS-2, and U2OS, have additionally served as models for evaluating initial cell adhesion, cytoskeletal tension, and matrix mineralization [71,72,73,74,75].

#### 3.3.2. Adhesion, Spreading, and Morphological Dynamics

The early biological responses of osteoblasts, from initial adhesion and spreading to overall morphogenesis, are strongly influenced by the specific topography of the implant. On smooth or polished Y-TZP surfaces, osteoblasts generally adopt a highly stretched, planar morphology characterized by a widely spread actin cytoskeleton [51,62,73]. Conversely, micro-roughened topographies frequently lead to morphologic irregularities, a less-expanded planar cytoskeleton, and reduced overall cell spreading areas [51,62,68,73]. However, despite this restricted horizontal spreading, the actual quantity of initially attached cells within the first 24 h is significantly higher on rough zirconia than on smooth surfaces [63]. These roughened topographies compensate for the reduced horizontal spreading area by promoting deep mechanical interlocking, allowing osteoblasts to firmly anchor their filopodia within the porous substrate [68]. Importantly, the strength of this initial cell-material binding, driven by the expression of focal adhesion kinases (FAK) and vinculin, is dictated entirely by the surface topography (rough vs. smooth) rather than the underlying implant material itself (zirconia vs. titanium) [69]. Furthermore, applying defined geometric micro-patterns can induce a strong “contact-guided” effect, causing osteoblasts to physically align their cytoskeletons parallel to the structural channels and establish highly ordered intercellular connections [53,61].

#### 3.3.3. Proliferation vs. Osteogenic Differentiation

During the osseointegration timeline, a well-established inverse relationship often exists between early cell proliferation and late-stage differentiation. In short-term culture periods, initial cellular attachment and proliferation on zirconia remain quantitatively comparable to standard titanium [54]. However, depending on the topography, initial growth rates tend to be higher on smooth Y-TZP surfaces compared to roughened topographies [51,62]. Over extended culture periods, the proliferation rates on both smooth and micro-roughened zirconia equalize and remain quantitatively comparable to those on standard titanium surfaces [39]. Despite the initial delay in spreading and proliferation, increased surface roughness directly correlates with enhanced long-term osteogenic differentiation. Micro-roughened topographies promote accelerated cell maturation and bone matrix mineralization compared to smooth surfaces by upregulating late differentiation markers such as osteocalcin [51,62,68], while correspondingly downregulating early markers like alkaline phosphatase over time [68].

#### 3.3.4. Impact of Specific Surface Modifications on Osteoblast Behavior

**Topographical and Structural Modifications:** Altering the physical landscape of zirconia strongly influences cellular dynamics. Standard subtractive micro-roughness significantly upregulates the expression of focal adhesion integrins α5 and β1, enhancing the initial mechanical attachment of osteoblasts [63]. When directly comparing subtractive methods, double acid-etched zirconia stimulates statistically higher expressions of key bone matrix proteins (Type I Collagen and Osteopontin) compared to identically etched titanium [52]. Similarly, sandblasted zirconia promotes a significantly higher rate of osteoblast migration (72% healing in scratch assays) compared to sandblasted titanium (51%), driven by the formation of denser intercellular filopodia contacts [59]. Specialized heat treatments can further refine these subtractive methods by altering the underlying crystallography. Rohr et al. (2020) [73] demonstrated that osteoblast viability correlates linearly with the tetragonal phase ratio of the zirconia surface; applying a heat treatment to recover the tetragonal phase (which is often lost to the monoclinic phase during grinding) significantly improves osteoblast viability [73]. Additionally, specialized sequential heat treatments create mild, nano-micro curved hills that enable tight cellular anchorage without triggering the intracellular stress, specifically ROS and ATP depletion, that is typically provoked when cells attempt to phagocytize sharp-edged features [74].

**Laser Ablation and Micro-Patterns:** When specific geometric micro-patterns are introduced via femtosecond or CO_2_ laser ablation, the stacked groove structures provide a larger 3D spatial area that drives extensive surface coverage at early, as well as rapid proliferation at later culture stages [53,61] and strongly upregulates key osteogenic regulators like Runx2, alkaline phosphatase, and osteopontin [61]. Conversely, Nd:YAG laser-textured grooves have not consistently shown superior osteoblast proliferation or spreading compared to standard sandblasting [64,65].

**Macroscopic Porosity and Additive Manufacturing:** Generating macroscopic 3D porosity via traditional powder metallurgy techniques utilizing sacrificial porogens (such as polyethylene particles) creates a complex 3D spatial network that optimizes nutrient circulation and accelerates spatial proliferation [55]. Furthermore, altering topography via modern 3D additive manufacturing provides unique structural benefits based on the printing technology. The inherent layer-by-layer micro-topography produced by Fused Filament Fabrication (FFF) mitigates secondary cellular detachment, supporting steady proliferation and significantly elevating late-stage osteocalcin expression [67]. Alternatively, zirconia fabricated via stereolithography (SLA/DLP) yields significant cell viability, elevated alkaline phosphatase activity, and Type I collagen expression comparable to or better than conventional sandblasted and acid-etched titanium [60].

**Surface Hydrophilicity and Wettability:** Modifying the surface energy of zirconia via non-thermal plasma treatments (such as O_2_, N_2_, or Ar/CF4) modifies surface chemistry by removing hydrocarbon contaminants and maximizing wettability and hydrophilicity [56,70,71]. While these treatments substantially improve initial contact angles, studies show that the underlying physical topography remains the primary driver of osteoblast morphology [70]. Nevertheless, dual-treated surfaces (e.g., combining sandblasting with Ar/CF4 plasma etching) introduce unique sub-micron features that support initial attachment with well-organized actin filaments comparable to standard titanium [71]. Most importantly, the chemical activation and hydrophilicity gained from plasma etching drives a significantly increased osteogenic response, yielding more favorable early osteoblast differentiation, elevated alkaline phosphatase activity [71], and calcium nodule deposition (especially with N_2_ plasma) [56,71].

**Biofunctional Coatings:** Because bare zirconia is inherently bioinert, it can occasionally restrict spontaneous cell spreading [56]. Functionalizing the surface with bioactive components successfully overcomes this limitation, but requires careful engineering to ensure coating stability. For instance, utilizing a low-temperature sol–gel method (800 °C) to coat HA prevents the formation of calcium zirconate by-products that normally weaken high-temperature coatings [57]. Alternatively, utilizing chemical covalent bonding (via APTES and succinic acid) to immobilize nano-HA and type I collagen prevents the bioactive layer from physically detaching or diffusing away in the body [58]. This covalent immobilization of collagen enables specific integrin-receptor recognition through RGD sequences, increasing adherent osteoblasts, cytoskeletal spreading, and robust filopodia extension [58]. Ultimately, these bioactive HA and collagen modifications significantly improve osteoblast spreading and proliferation rates to match robust titanium profiles [57,58], while enhancing alkaline phosphatase activity and in vitro bone matrix mineralization [58]. In contrast, Mineral Trioxide Aggregate (MTA) coatings on laser-textured Y-TZP surface result in lower initial viability, but strongly drive advanced osteoblast differentiation and osteocalcin secretion in the few adhering cells [66], while carbon-based C60 films maintain baseline cell viability and proliferation without impeding overall osteogenesis [38].

**The Inflammatory Microenvironment and Pathogenic Challenge:** While specific surface modifications directly dictate osteogenesis, the success of these processes is ultimately governed by the surrounding immune and microbiological microenvironment. The presence of early pro-inflammatory cytokines (such as TNF-α) strongly activates the NF-κB signaling pathway in osteoblast-like cells (MG-63) on both zirconia and titanium with sandblasted and acid etched surfaces, which actively suppresses their overall metabolic activity [72]. Furthermore, in post-operative scenarios where the soft tissue seal is breached by anaerobic sub-gingival pathogens (e.g., *P. gingivalis*, *P. intermedia*), zirconia demonstrates superior biological resilience compared to titanium. In in vitro co-culture models, adhering osteoblasts withstand pathogenic challenges significantly more effectively on zirconia than on pure titanium, maintaining robust surface coverage and adhesion regardless of the underlying surface roughness [75]. This highlights the critical need for advanced surface modifications that not only promote osteoconductivity but also actively resolve macrophage-induced inflammation and resist pathogenic displacement to ensure unhindered long-term bone integration.

#### 3.3.5. Key Remarks

Successful osseointegration of Y-TZP implants requires a strategic engineering balance that overcomes the material’s inherent bioinertness while addressing complex topographical, biochemical, and microbiological demands. Although smooth zirconia surfaces initially facilitate rapid cell spreading and proliferation, the introduction of advanced micro- and nano-topographies; whether through subtractive treatments, laser ablation, or additive manufacturing, is essential for establishing deep mechanical interlocking, maximizing initial cell attachment, and driving long-term osteogenic differentiation without provoking detrimental intracellular oxidative stress. As physical topography alone is often insufficient to maximize bioactivity, structural modifications must frequently be coupled with chemical activation, such as plasma treatment or covalent immobilization of bioactive ligands, to accelerate bone matrix mineralization. These precisely engineered zirconia interfaces confer biological resilience, enabling adherent osteoblasts to maintain adequate surface coverage and remain stable even when challenged by pro-inflammatory cytokines and invading subgingival pathogens.

It is of importance to note the predominance of osteosarcoma-derived cell lines (MG-63, SaOS-2, and U2OS) and murine preosteoblasts (MC3T3-E1) in the available evidence. Because these models differ from primary human osteoblasts in their proliferative and differentiation behavior, findings obtained using them should not be directly extrapolated to the clinical setting. Comparisons across studies are further complicated by inconsistent surface-characterization protocols. Some studies report two-dimensional roughness parameters (Ra), whereas others report three-dimensional parameters (Sa), often measured using different instruments, scan areas, resolutions, and experimental conditions. Consequently, the available data do not permit reliable identification or comparison of roughness thresholds associated with optimal osteogenic responses. Among the approaches reviewed, favorable osteogenic outcomes across independent studies were associated with plasma treatment, particularly when combined with subtractive surface roughening, as well as with hydroxyapatite coatings and the covalent immobilization of collagen or RGD-containing peptides. In contrast, the evidence regarding laser-textured surfaces remains heterogeneous. Although femtosecond- and CO_2_-laser treatments have generally produced more favorable outcomes than those reported for Nd:YAG-modified surfaces, the absence of standardized, direct head-to-head comparisons prevents firm conclusions regarding the relative superiority of individual laser systems.

### 3.4. Fibroblasts

Fibroblasts are the principal architects of connective tissue, responsible for producing and remodeling the ECM. Their synthesis of Type I collagen fibers directly determines the firmness of the gingiva. Fibroblasts also contribute to the formation of the mucosal seal, a biological barrier between the oral environment and the alveolar bone [66,76]. This seal acts as a protective barrier preventing the infiltration of exogenous bacteria and noxious substances into the implant–tissue interface [66,77]. Without firm fibroblast attachment to the abutment surface, the biological width may recede, leading to apical migration of the junctional epithelium, pocket formation, and inflammation-driven bone loss [78,79]. In contrast to the perpendicular Sharpey’s fibers anchored in the cementum of natural teeth, peri-implant connective tissue fibers are typically oriented parallel to the implant surface, rendering the attachment inherently more vulnerable [66,80]. Consequently, strong adhesion and high bioactivity of human gingival fibroblasts (HGFs) are critical to compensate for this structural weakness and maintain a tight tissue seal [80].

#### 3.4.1. Cell Lines Utilized in Current Evidence

The biological response of soft tissue to Y-TZP has been extensively characterized using a variety of cell models. Primary HGFs are the most frequently employed due to their clinical relevance as the dominant cell type in gingival connective tissue, used either as primary isolates [76,77,79,80,81,82,83,84,85,86] or as commercially available lines [78,87,88,89]. Established immortalized lines, including HGF-hTERT, iHGF, and HGF-1, are used to ensure experimental reproducibility [64,66,90,91]. Mouse-derived fibroblast lines, including L929 and Balb/c 3T3, serve as sensitive models for assessing cytotoxicity and metabolic responses on modified surfaces [92,93,94].

#### 3.4.2. Adhesion, Spreading, and Morphological Dynamics

Fibroblast behavior on zirconia is characterized by a so-called “rugophobic” nature, reflecting a general preference for smoother surfaces (Sa < 0.2 µm) [76,78]. On highly polished or translucent Y-TZP, fibroblasts typically exhibit an elongated, spindle-shaped morphology with extensive actin filament networks and multiple filopodial extensions [90], indicative of strong mechanical stabilization and high cellular affinity [78,90]. On heterogeneous or excessively rough topographies, fibroblasts may appear more rounded and require longer to achieve initial attachment [78,82,93]. Comparative analysis of machined and polished zirconia surfaces suggests that the molecular response of HGFs is more sensitive to the duration of surface contact than to the initial topographical finish alone. Regardless of surface roughness or material type (titanium vs. ceramic), HGFs exhibit a consistent time-dependent upregulation of vinculin, paxillin, tensin, and FAK [85]. An important characteristic of fibroblast behavior on zirconia is contact guidance, a phenomenon whereby cells align their cytoskeleton parallel to micro-scale grooves or machining tracks [78,81,84]. This alignment is mediated by focal adhesions (FAs), whose number and area serve as quantitative indicators of adhesion strength [76]. The maturation of the fibroblast–implant bond is a time-dependent process intrinsically linked to the expression of focal adhesion linker proteins (FALPs). Adhesion strength, measured by resistance to mechanical detachment via ultrasonic waves, increases significantly between day 1 and day 3 of culture, paralleling the biological maturation of the focal adhesion complex. Specifically, mRNA and protein expression of vinculin and paxillin in HGFs increases significantly from day 1 to day 5, identifying these proteins as primary mediators of mucosal seal stability at the transmucosal interface [85].

#### 3.4.3. Proliferation and Metabolic Activity

Although fibroblasts do not undergo osteogenic differentiation, their functional performance is commonly evaluated across three parameters: viability, proliferation, and ECM production, particularly Type I collagen [64,77,87]. Ultra-translucent zirconia variants containing a higher proportion of the cubic phase (~52.3%) support significantly greater HGF viability than traditional variants, likely due to enhanced chemical stability [89]. However, clinical finishing procedures such as glazing can suppress proliferation through increased roughness and deposition of elements including silicon and potassium [89]. Recent metabolic analysis using two-photon-excited fluorescence lifetime imaging microscopy (FLIM) has revealed that fibroblasts on porous zirconia may enter a state of hypoxia characterized by a shift toward glycolysis (free NAD(P)H), even when proliferation rates appear normal [94].

#### 3.4.4. Impact of Specific Surface Modifications on Fibroblast Behavior

**Topographical and Structural Modifications:** Milled zirconia surfaces (Sa ≈ 0.36 μm) have demonstrated superior cell attachment and proliferation compared to highly polished specimens, suggesting that fine irregularities can increase the surface area available for protein adsorption [84]. However, when roughness exceeds the sub-micron scale (e.g., Ra ≈ 30.93 μm), adhesion is significantly impaired [93].

**Laser Ablation and Micro-Patterns:** Laser-textured grooves (typically 10–120 μm wide) facilitate contact guidance but do not always outperform traditional sandblasted and acid-etched surfaces in terms of total cell viability. The research conducted by da Cruz et al. (2022) [64] demonstrated that macrotopographic patterns do not provide a superior biological response for either hard or soft tissue cells when compared to the standard by sandblasting and acid etching microtopography. While for bone cells, traditional milling was better than using a laser, soft tissue cells (fibroblasts) responded about the same regardless of which texturing method was used [64].

**Macroscopic Porosity:** The presence of macroscopic porosity may limit oxygen transport within the material, prompting cells to rely more heavily on glycolytic metabolism as a compensatory response diffusion [94].

**Biofunctional Coatings:** Covalent immobilization of ECM proteins like fibronectin and laminin using silane crosslinkers (e.g., APDS) significantly increases focal adhesion counts and cell area [80,83]. Coatings with MTA have also been shown to induce fibroblast proliferation compared to native textured surfaces [66].

**The Inflammatory Microenvironment and Pathogenic Challenge:** Antibacterial modifications, such as silver nanoparticle (AgNP) coatings or zinc oxide (ZnO) doping, can inhibit pathogen growth [91,92]. While silver is non-cytotoxic at densities of 32 μg/cm^2^, ZnO exhibits a dose-dependent toxicity where concentrations above 60% may reduce fibroblast viability due to nanoparticle uptake [91,92]. Modified zirconia surfaces do not inherently promote inflammation; HGFs cultured on Y-TZP, Ti, and PEEK show statistically similar levels of inflammatory gene expression [86]. Furthermore, neither Y-TZP nor PIC hybrid ceramics affect the ability of cells to produce their own extracellular matrix, despite the introduction of polymer components [88].

#### 3.4.5. Key Remarks

The available evidence indicates that fibroblast behavior on zirconia is influenced by an interplay of surface chemistry, energy, and topography. The question of optimal surface roughness remains unresolved: while fibroblasts are generally considered to favor smoother surfaces, certain milled micro-topography patterns may enhance attachment by increasing the protein-binding surface area. A notable advancement in surface optimization is the development of biofunctional protein coatings, specifically, fibronectin and laminin covalently immobilized via silane crosslinkers, that are both effective at promoting adhesion and experimentally stable, resisting hydrolysis for up to 21 days and remaining stable against enzymatic and mechanical challenge. These properties suggest the ability to withstand the fluctuating conditions of the oral environment. Additionally, the metabolic shifts toward hypoxia observed on certain ceramic structures underscore the need for future research to move beyond simple viability assays and incorporate functional metabolic profiling to better predict long-term clinical performance.

The available evidence on fibroblast responses reveals several unresolved methodological inconsistencies. The optimal roughness threshold for HGF attachment remains contested: while the rugophobic preference for smoother surfaces (Sa < 0.2 µm) is the most frequently reported finding, results from milled zirconia at Sa ≈ 0.36 µm suggest that a degree of controlled micro-irregularity may enhance protein adsorption and attachment, indicating that the relationship between roughness and HGF response is not strictly linear. Comparison across studies is further complicated by the use of both primary HGF isolates and immortalized lines, which differ in adhesion dynamics and ECM production capacity. The notable finding is that covalent immobilization of fibronectin and laminin via silane crosslinkers enhances focal adhesion formation and cell area across multiple independent studies. However, the durability of these coatings under the cyclic mechanical and enzymatic challenge of the oral environment remains incompletely characterized, and clinical validation of these in vitro findings is lacking.

### 3.5. Epithelial Cells

The long-term success of a dental implant relies heavily on establishing a resilient mucosal seal, where epithelial cells rapidly migrate and proliferate to form the peri-implant epithelium (PIE), the primary physical barrier against the microbe-rich oral environment [95]. In a healthy state, this attachment mimics the natural junctional epithelium, anchoring to the implant surface via a specialized basal lamina rich in Laminin-332, which binds to cellular hemidesmosomes through integrins α6 and β4 [96]. However, the PIE is inherently more fragile and frequently lacks this stringent basal lamina-mediated sealing, making the implant highly susceptible to bacterial biofilm invasion and peri-implantitis [97]. Because epithelial cells biologically prefer smooth or nanostructured surfaces to facilitate rapid migration and barrier formation, Y-TZP has emerged as a highly promising abutment material [95]. Y-TZP offers favorable aesthetics, reduces plaque accumulation, and actively promotes of hemidesmosomal attachment assembly [98].

#### 3.5.1. Cell Lines Utilized in Current Evidence

To elucidate the mechanisms underlying soft-tissue integration, a range of established in vitro models has been employed to evaluate the epithelial response to Y-TZP surfaces. The most widely used primary cell models include human oral keratinocytes (HOK) and human gingival keratinocytes (HGK) [95,99]. To overcome the limited lifespan of primary cells, researchers rely extensively on immortalized gingival keratinocyte lines, including OKG4/bmi1/TERT, TERT2/OKF6, spontaneously immortalized human gingival keratinocytes (GK), and the OBA-9 human gingival epithelial cell line [96,97,98,100,101]. Additionally, advanced three-dimensional peri-implant mucosal models (3D-PIMM) utilizing TR146 epithelial cancer cells have served as robust models to evaluate functional endpoints such as tissue-abutment interface contours [102].

#### 3.5.2. Adhesion, Spreading, and Morphological Dynamics

Epithelial cell adhesion and early morphological spreading are governed by the topography and wettability of the Y-TZP surface [96,100]. Unmodified Y-TZP is not inherently hydrophobic. It has been observed to exhibit a higher water contact angle than Ti in certain comparative assays, though both materials generally demonstrate comparable hydrophobic tendencies [98,100]. Despite this, epithelial cells successfully attach to and spread across Y-TZP, demonstrating superior cell coverage compared to control substrates such as bovine enamel [100]. Topography plays a defining role in morphological adaptation: on smooth or polished Y-TZP, keratinocytes adopt a flattened, pavement-like arrangement with extensively spread actin cytoskeletons and mature focal adhesion plaques localizing vinculin at the cell periphery [95,96]. Conversely, increased surface roughness significantly restricts horizontal cellular expansion. On rougher Y-TZP surfaces, such as those subjected to sandblasting and acid-etching, keratinocytes become physically confined within micrometer-scale topographical features, frequently adopting a stationary morphology rather than spreading fully [96].

#### 3.5.3. Migration, Proliferation, Differentiation, and Immunology

Establishing a functional peri-implant barrier requires a coordinated balance of cellular migration, proliferation, and differentiation. Y-TZP actively supports a highly motile epithelial phenotype, permitting keratinocytes to form KRT5 perinuclear cages indicative of active motility on smooth surfaces [96]. Regarding proliferation, evidence is mixed: some studies report lower early metabolic activity on Y-TZP compared to Ti [98] or control substrates [99], whereas others demonstrate that keratinocyte viability and cell density on smooth Y-TZP increase rapidly, matching or exceeding Ti over extended culture periods [95,96]. Smooth Y-TZP appears to efficiently direct molecular differentiation toward the junctional epithelium (JE) lineage, upregulating critical hemidesmosome components (integrins α6 and β4) compared to Ti [98] and inducing JE-specific differentiation markers including keratin 19 (KRT19) and odontogenic ameloblast-associated protein (ODAM) [96]. From an immunological perspective, the successful differentiation and structural integrity of this JE layer are vital, as it forms the primary physical barrier against microbial invasion and facilitates physiological defense by permitting the passage of polymorphonuclear cells (PMNs) and lymphocytes into the peri-implant region [97].

#### 3.5.4. Impact of Specific Surface Modifications on Epithelial Cell Behavior

To overcome the inherent bioinertness of zirconia and accelerate soft-tissue barrier formation, various surface modifications have been evaluated in vitro.

**Topographical refinement:** Modification of Y-TZP at the macroscopic level influences integration outcomes. Highly polished Y-TZP surfaces are shown to enhance initial epithelial cell adhesion and uniquely permit the widespread deposition of Laminin-332, a critical basement membrane protein, across the entire surface. In contrast, rough, unpolished Y-TZP seem to restrict Laminin-332 secretion to localised topographic recesses [96].

**UV photofunctionalization:** Ultraviolet (UV) light treatment alters the surface chemistry of Y-TZP without modifying its physical topography. When evaluated in a 3D-PIMM, UV photofunctionalization significantly increased cellular migration toward the material and promoted the development of a tight, non-pocket soft-tissue contour, representing a favorable configuration for the prevention of bacterial ingress [102].

**Bioactive coatings:** The application of sol–gel-derived, nanoporous titanium dioxide (TiO_2_) coatings to Y-TZP surfaces improves early keratinocyte attachment and cytoskeletal spreading. Furthermore, this bioactive TiO_2_ coating directly induces the localised expression of key adhesion proteins, elevating the deposition of Laminin γ2 and integrin α6 on zirconia [101].

**Peptide functionalization:** Owing to the chemical inertness of pristine Y-TZP, conventional peptide applications often fail; however, an electrophoretic fusion (EPF) method employing a phosphonic acid (PA) linker successfully immobilizes Protease-Activated Receptor 4 (PAR4) peptides directly onto the Y-TZP surface. This biomimetic PAR4 functionalization actively induces local platelet aggregation and the release of platelet-derived growth factor-AB (PDGF-AB). Consequently, it was reported that this platelet-rich environment drives human gingival epithelial cells to form dense, elongated colonies that actively secrete Laminin-5, establishing basal lamina-mediated attachment to the ceramic [97].

#### 3.5.5. Key Remarks

The reviewed in vitro evidence identifies a fundamental incompatibility between osseointegrative and mucointegrative surface requirements: the micro-roughness optimized for osteoblast interlocking consistently restricts epithelial cell spreading and physically entraps cells within topographic features. Despite this complexity, the distinction between simple focal adhesion and hemidesmosome-mediated attachment remains critically important. Optimizing Y-TZP for mucointegration requires a hybrid strategy. First, macroscopic surface smoothness, achieved preferentially through polishing, is essential to facilitate initial epithelial coverage, active motility, and high terminal cell densities. Second, targeted surface modifications are additionally indispensable for the chemical promotion of bioinduction and hemidesmosomal assembly. As demonstrated by nanoporous TiO_2_ coatings, UV photofunctionalization, and biomimetic PAR4 peptide immobilization, enhancement of surface chemistry can substantially amplify the soft-tissue response, mitigating the inherent bioinertness of pristine Y-TZP, promoting hemidesmosome assembly, and yielding a tight peri-implant biological seal capable of resisting bacterial penetration.

The epithelial cell evidence base is the most limited among the five cell types reviewed, both in the number of available studies and in the range of surface modifications evaluated. The finding that smooth or polished Y-TZP surfaces favor keratinocyte spreading, Laminin-332 deposition, and hemidesmosomal differentiation is supported by multiple studies and represents the most clinically actionable conclusion in this section. However, the majority of studies employ immortalized cell lines under static monoculture conditions, which do not capture the dynamic mechanical environment of the transmucosal abutment in vivo. The hemidesmosomal differentiation markers reported (KRT19, ODAM, integrins α6/β4) are promising indicators of junctional epithelium formation, but their relationship to clinical soft-tissue sealing outcomes (mucosal recession, probing depth, peri-implant mucosal health) has not been established in human trials.

## 4. Summary of Current Evidence

The reviewed literature highlights a fundamental conflict between “rugophobic” and “rugophilic” cellular responses to surface topography. This cell specificity has long been recognized: epithelial cells generally show less favorable attachment and spreading on acid-etched and sandblasted surfaces than on smooth, polished or optically flat surfaces, whereas fibroblasts may adhere comparably to rougher, machined and smooth surfaces [103,104]. Within the zirconia-focused literature reviewed here, subtractive microtopographies enhance osteoblast attachment and osteogenic activity [68] and promote MSC differentiation [42], but may restrict epithelial-cell spreading [96,98]. Fibroblast responses appear more heterogeneous, ranging from more favorable behavior on smooth surfaces to enhanced attachment on finely irregular milled surfaces [76,78,84,93]. Increased micro-roughness may also favor acute pro-inflammatory M1 macrophage polarization [37], while particular rough or microstructured surfaces have been associated with early hypoxic signaling and a metabolic shift toward glycolysis in fibroblasts [94]. Collectively, these observations indicate that no single roughness range can be considered universally optimal across the peri-implant interface. Moreover, because roughness is closely associated with differences in surface chemistry, wettability, feature geometry and topographical scale, the available evidence does not permit the definition of a universal optimal roughness threshold even for an individual cell type. Furthermore, aggressive mechanical protocols such as sandblasting, or nanosecond laser texturing used to generate osteoconductive roughness, may introduce surface flaws and microcracks that serve as nucleation points for LTD, potentially compromising the hydrothermal and long-term mechanical stability of Y-TZP [46,48]. The prevailing biomaterial paradigm, largely derived from titanium research, holds that maximizing surface hydrophilicity resolves roughness-induced inflammation. However, evidence suggests that nanoscale physical topography may exert a stronger influence than surface free energy on macrophage polarization under certain experimental conditions [36], indicating that straightforward extrapolation from titanium literature to zirconia is limited and warrants dedicated investigation.

The reviewed literature reveals a clear developmental trajectory in surface engineering strategies, from passive characterization of standard subtractive topographies toward increasingly precise and biologically instructive interventions. Earlier efforts focused predominantly on basic attachment and viability responses to sandblasted or acid-etched surfaces, evaluating the mechanical interlocking of macrophages, osteoblasts, and mesenchymal stem cells, while documenting the topographical limitations and restricted spreading encountered by rugophobic fibroblasts and epithelial cells.

More recent work has shifted toward architecturally controlled modifications addressing the specific physical requirements of multiple cell lineages. Femtosecond laser microgrooves, for instance, are now employed not only to guide osteoblast alignment [53], but also to promote rapid anisotropic migration in mesenchymal stem cells and provide low-periodicity nanostructures that mechanically incapacitate bacteria and protect stem cell integrity [44]. Similarly, additive manufacturing approaches generate macro-porous and layer-by-layer topographies that support sustained osteoblast proliferation and metabolic activity [60,67]. While porous zirconia architectures remain biocompatible for fibroblasts, it may induce glycolytic metabolic adaptation because of reduced oxygen diffusion [94].

In parallel, immunomodulatory and bio-inductive surface functionalization has advanced from passive chemical modifications toward the targeted deployment of bioactive films designed to manipulate specific cellular responses. For immune regulation, fullerene C60 coatings and time-controlled cerium dioxide nanoparticle bilayer systems selectively modulate macrophage intracellular signaling pathways, including TLR4/MyD88/NF-κB, to actively resolve inflammation [34,38]. For soft-tissue mucointegration, bio-instructive strategies have evolved to actively recruit fibroblasts and epithelial cells, employing UV photofunctionalization and the biomimetic electrophoretic immobilization of specific agents such as PAR4 peptides to promote the assembly of a tight hemidesmosomal seal [97,102]. Concurrently, advanced dual-peptide platforms combining adhesive (RGD) and osteogenic (DWIVA) motifs are employed to chemically maximize focal adhesion and terminal differentiation of mesenchymal stem cells [43]. Preferred surface characteristics for each cell type, associated biological responses, modification strategies, and key design challenges are summarized in Table 1.

Taken together, these developments reflect a broader shift in the field toward surfaces designed to actively guide the interdependent responses of all critical host cell populations at the molecular level, although the long-term in vivo relevance of these in vitro findings remains to be established.

## 5. Limitations and Future Directions

A key limitation of this review is its strict methodological scope, focusing exclusively on in vitro cell culture studies and explicitly excluding in vivo animal models and clinical human trials. While the reviewed literature provides valuable mechanistic insights at the molecular level, its reliance on static monocultures or simplified co-cultures fails to fully replicate the complexity of the post-operative peri-implant environment. The true clinical scenario involves the simultaneous and interdependent activity of immune cells, bone-forming cells, soft-tissue components, and invading oral pathogens. Current static in vitro models cannot adequately simulate systemic immune responses, cyclic biomechanical loading, or fluid shear stress. Additionally, as this is a narrative review, no formal risk-of-bias assessment or systematic screening records were generated. Individual in vitro studies vary considerably in cell source (immortalized lines vs. primary cells), replicate number, and statistical rigor.

A further limitation that warrants explicit acknowledgment is the substantial gap between in vitro biological performance and clinical implant outcomes. Several surface modifications reviewed, including femtosecond laser texturing, dual-peptide functionalization, and cerium dioxide bilayer coatings, demonstrate encouraging cellular responses under controlled laboratory conditions. However, the transition from in vitro efficacy to clinical benefit is rarely linear, and the vast majority of modifications discussed in this review lack supporting data from well-designed in vivo animal studies and long-term randomized clinical trials. Observed differences in ALP activity, osteocalcin expression, or cytokine secretion between surface groups, while statistically significant, may not correspond to clinically meaningful improvements in implant survival, bone-to-implant contact, or peri-implant tissue health. Readers should therefore interpret the in vitro findings presented here as mechanistic evidence pointing toward promising research directions, rather than as direct evidence of clinical superiority.

The interpretation of the available evidence is further challenged by considerable methodological heterogeneity across the studies included in this review. Cell models vary widely, from murine cell lines (RAW 264.7, MC3T3-E1, L929) to primary human isolates and immortalized lines. Each of the cell models mentioned features distinct biological characteristics that affect the magnitude and direction of observed responses. Culture conditions, including medium composition, serum concentration, passage number, and culture duration, are rarely standardized across studies. Additionally, surface characterization methods are inconsistent with different roughness parameters reported, measured using different profilometry instruments and evaluation lengths, making direct numerical comparisons unreliable. Bioactive coating studies are particularly difficult to compare, as loading concentrations, immobilization protocols, and stability assessments differ substantially between laboratories. This heterogeneity underscores the need for the field to adopt standardized reporting frameworks for both surface characterization and cell culture methodology, to enable meaningful synthesis of in vitro zirconia research in the future.

Beyond the surface modification strategies reviewed, several emerging research directions hold particular promise for the next generation of zirconia implant design. Artificial intelligence and machine learning approaches are increasingly being applied to accelerate biomaterial discovery and design across metals, ceramics, and implantable devices, reducing reliance on traditional trial-and-error approaches with data-driven prediction [105], a principle that could similarly enable high-throughput computational screening of zirconia surface parameters to predict cell-specific biological responses prior to fabrication. Multifunctional antimicrobial coatings represent another important direction, and have already been demonstrated directly on zirconia: a layer-by-layer chlorogenic acid-chitosan conjugate coating applied to yttria-stabilized zirconia was shown to simultaneously enhance osteogenic differentiation and antibacterial activity in a single multifunctional coating system [106]. Extending such bifunctional strategies to also incorporate immunomodulatory control of macrophage polarization represents a logical next step toward coatings that address osseointegration, immune balance, and antibacterial resistance simultaneously. Furthermore, experimental advances in additive manufacturing have enabled zirconia dental implants to be fabricated with spatially controlled surface features. Additively manufactured zirconia implants have been shown to seamlessly integrate a rough, osteoconductive porous surface layer with a dense implant core within a single build [107]. Extending this capability toward a full gradient, transitioning to smoother, bio-inductive topographies in the transmucosal zone, could enable a single, seamlessly fabricated zirconia component optimized for both bone and soft-tissue interfaces. Nanostructured surfaces and immunomodulatory biomaterials are emerging as potentially complementary approaches in zirconia implant engineering. Available evidence indicates that nanoscale surface characteristics can influence cell adhesion and migration [44], while certain nanostructured zirconia surfaces may also modulate macrophage polarization, with downstream effects on fibroblast behavior [36]. Fullerene films and cerium dioxide-based coatings have likewise demonstrated anti-inflammatory effects, with some systems also exhibiting antibacterial or osteogenic properties [34,38]. Collectively, these findings suggest the potential for multifunctional zirconia surfaces combining topographical and immunomodulatory cues; however, further preclinical and clinical validation is required.

Future implant surface engineering will likely continue to transition from passive subtractive topographies toward increasingly precise, multi-scale, and biologically instructive interventions. To resolve the topographical conflict between osseointegration and soft-tissue mucointegration, the development of zoned implant modifications appears promising. Macroscopically smooth transmucosal regions could benefit from bio-inductive surface properties, such as UV photofunctionalization or biomimetic peptide immobilization (e.g., PAR4), to actively promote assembly of the hemidesmosomal apparatus (integrins α6/β4 and Laminin-332) required for a tight junctional epithelial seal. The bone-anchored regions, meanwhile, may benefit from advanced manufacturing approaches such as ultrashort-pulsed (femtosecond) laser ablation coupled with post-laser annealing, enabling the creation of precise micro- and nano-topographies that guide osteoblast alignment while avoiding the thermal damage and phase alterations associated with LTD.

To overcome the early bioinertness of zirconia and favor host cell colonization over bacterial adhesion in the “race for the surface,” future strategies should build on active immunomodulatory and antibacterial functionalizations. Promising directions include time-controlled smart coatings, such as bilayer cerium dioxide nanoparticle systems or fullerene (C60) films, that selectively attenuate local pro-inflammatory signaling pathways (e.g., TLR4/MyD88/NF-κB), alongside low-periodicity laser patterns (~3 µm) that reduce bacterial adhesion and aggregation while preserving host cell integrity.

Methodologically, research is expected to progress beyond single-variable viability assays and sequential conditioned-media studies. Future investigations should prioritize functional metabolic profiling, such as fluorescence lifetime imaging microscopy (FLIM), to assess cellular states including hypoxia on novel porous architectures. The natural progression in the field lies in transitioning from static in vitro models to systems-level tissue modeling. Microfluidic organ-on-a-chip platforms and three-dimensional bioprinted peri-implant mucosal models (3D-PIMM) will enable researchers to more accurately recapitulate the bone–transmucosal–epithelial continuum by simulating fluid shear stress, dynamic pathogen challenges, and spatially organized multicellular populations, substantially strengthening the translational bridge between in vitro mechanistic evidence and the clinical reality of Y-TZP peri-implant healing.

In conclusion, the current literature demonstrates a progressive shift from designing zirconia surfaces that are merely osteoconductive toward surfaces capable of actively directing cell-specific functions through topographical and biochemical cues. Future advances are therefore likely to depend not on increasing surface complexity alone, but on tailoring surface characteristics to elicit cell-specific biological responses while preserving the mechanical stability and long-term reliability of zirconia.

## Figures and Tables

**Table 1:** Cell-specific determinants of Y-TZP implant integration: surface preferences, biological responses, modification strategies, and design challenges across the peri-implant healing cascade.

Cell Type	Preferred Surface Characteristics	Primary Biological Response	Surface Modifications Strategy	Design Challenge
Macrophages	Smooth nanostructured surfaces with high bioactivity	M2 polarization, reduced inflammatory cytokine production, osteoimmunomodulation	Fullerene (C60) coatings, CeO_2_/PDA smart coatings	Minimize pro-inflammatory effects of micro-roughness while preserving osteogenic potential
Mesenchymal stem cells	Hierarchical micro-/nanotopography with biochemical guidance	Adhesion, migration, mechanotransduction and osteogenic commitment	SLA-like treatments, USP laser patterning, 3D scaffolds, peptide functionalization (RGD/DWIVA)	Promote osteogenic differentiation without compromising zirconia mechanical stability
Osteoblasts	Moderately rough microtopography with bioactive surface chemistry	Matrix mineralization, osteogenic differentiation and osseointegration	Sandblasting/acid etching, laser ablation, additive manufacturing (FFF/DLP), heat treatments, plasma treatments, HA/collagen/MTA coatings	Balance early proliferation with long-term differentiation while maintaining material stability
Fibroblasts	Smooth, hydrophilic, high-energy surfaces	Connective tissue attachment, collagen production and mucosal integration	ECM protein coatings (fibronectin/laminin), MTA coatings, AgNP/ZnO antibacterial doping	Excessive roughness compromises fibroblast adhesion and metabolic activity
Epithelial cells	Smooth or nanostructured surfaces with high surface energy	Rapid migration, hemidesmosome formation and epithelial sealing	UV photofunctionalization, nanoporous TiO_2_ coatings, PAR4 peptide functionalization via electrophoretic fusion	Surface properties favorable for epithelial sealing may conflict with osteoblast requirements

## Data Availability

No new data were created or analyzed in this study. Data sharing is not applicable to this article.

## Abbreviations

The following abbreviations are used in this manuscript:

3D-PIMM	Three-dimensional peri-implant mucosal models
AgNP	Silver nanoparticles
ALP	Alkaline phosphatase
APTES	(3-Aminopropyl) triethoxysilane
Ar	Argon
ATP	Adenosine triphosphate
BMZ	Blank-machined nanostructured zirconia
C60	Fullerene
CaP	Calcium phosphate
CeO_2_	Cerium dioxide
CF4	Carbon tetrafluoride
CO_2_	Carbon dioxide
DLP	Digital light processing
ECM	Extracellular matrix
FAK	Focal adhesion kinase
FALPs	focal adhesion linker proteins
FAs	Focal adhesions
FFF	Fused filament fabrication
FLIM	Fluorescence lifetime imaging microscopy
GK	Gingival keratinocytes
HA	Hydroxyapatite
hADMSCs	Human adipose-derived mesenchymal stem cells
HGFs	Human gingival fibroblasts
HGK	Human gingival keratinocytes
hMSCs	Human mesenchymal stem cells
HOK	Human oral keratinocytes
IL-10	Interleukin-10
IL-1β	Interleukin-1beta
IL-6	Interleukin-6
JE	Junctional epithelium
KRT19	Keratin 19
KRT5	Keratin 5
LIPSS	laser-induced nanoripples
LPS	Lipopolysaccharide
LTD	Low temperature degradation
MSCs	Mesenchymal stem cells
MTA	Mineral trioxide aggregate
N_2_	Nitrogen
NPs	Nanoparticles
O_2_	Oxygen
ODAM	Odontogenic ameloblast-associated protein
PA	Phosphonic acid
PAR4	Protease-activated receptor 4
PDA	Polydopamine
PDGF-AB	Platelet-derived growth factor-AB
PIE	Peri-implant epithelium
PMNs	Polymorphonuclear cells
Ra	Roughness Average
RGD	Arginine-glycine-aspartic acid motif
ROS	Reactive oxygen species
Sa	Average Surface Roughness
SGZ	Self-glazed zirconia
SLA	Stereolithography
SLA-like surfaces	Sandblasting followed by acid etching
TGF-β	Transforming growth factor-beta
Ti	Titanium
Ti-6Al-4V	Titanium alloy
Ti-Zr	Titanium-zirconium alloy
TiO_2_	Titanium dioxide
TLR4/MyD88/NF-κB	MyD88-dependent Toll-like receptor 4 (TLR4) signaling pathway
TNF-α	Tumor necrosis factor-alpha
USP	Ultrashort-pulsed
UV	Ultraviolet
Y-TZP	Yttria-stabilized tetragonal zirconia polycrystal
ZLA	Zirconia Large-grit Sandblasted and Acid-etched Surface
ZnO	Zinc oxide
ZrO_2_	Zirconia
